# Eutherians experienced elevated evolutionary rates in the immediate aftermath of the Cretaceous–Palaeogene mass extinction

**DOI:** 10.1098/rspb.2015.3026

**Published:** 2016-06-29

**Authors:** Thomas John Dixon Halliday, Paul Upchurch, Anjali Goswami

**Affiliations:** 1 Department of Earth Sciences, University College London, Gower Street, London WC1E 6BT, UK; 2 Department of Genetics, Evolution and Environment, University College London, Gower Street, London WC1E 6BT, UK

**Keywords:** mammals, evolutionary rate, extinction, radiation, Cretaceous, Palaeogene

## Abstract

The effect of the Cretaceous–Palaeogene (K–Pg) mass extinction on the evolution of many groups, including placental mammals, has been hotly debated. The fossil record suggests a sudden adaptive radiation of placentals immediately after the event, but several recent quantitative analyses have reconstructed no significant increase in either clade origination rates or rates of character evolution in the Palaeocene. Here we use stochastic methods to date a recent phylogenetic analysis of Cretaceous and Palaeocene mammals and show that Placentalia likely originated in the Late Cretaceous, but that most intraordinal diversification occurred during the earliest Palaeocene. This analysis reconstructs fewer than 10 placental mammal lineages crossing the K–Pg boundary. Moreover, we show that rates of morphological evolution in the 5 Myr interval immediately after the K–Pg mass extinction are three times higher than background rates during the Cretaceous. These results suggest that the K–Pg mass extinction had a marked impact on placental mammal diversification, supporting the view that an evolutionary radiation occurred as placental lineages invaded new ecological niches during the Early Palaeocene.

## 1. Background

The K–Pg mass extinction occurred 66 Ma and was the second largest extinction event in the history of life, exterminating 75% of terrestrial species [1]. It marks a perceived shift from non-avian dinosaur-dominated terrestrial vertebrate faunas [2] to ‘mammal-dominated’ faunas (despite a greater modern richness of birds) [3,4], and is therefore often considered the start of the so-called ‘Age of Mammals’ [5]. As there are no known unambiguous Mesozoic placental mammal fossils [6,7], but many Palaeocene placentals, the K–Pg boundary has been considered a turning point in mammal evolution, with an adaptive radiation that ultimately resulted in the present-day placental mammal diversity [8].

Most recent analyses dating the origin of Placentalia have used molecular clock or clock-like methods, and often recovered divergence estimates in the ‘mid’ or Early Cretaceous for both Placentalia and several of its subclades [9–12]. The earliest fossil placental mammals should therefore be Cretaceous, but none is known, despite the existence of many Cretaceous eutherians. Indeed, a recent phylogenetic analysis comprising Palaeocene and Cretaceous placentals demonstrated that in every topology where extant ordinal relationships were constrained, no Cretaceous eutherian was resolved within crown-group Placentalia [13]. If these relationships of Cretaceous eutherians to the crown group are accurate, either the placental fossil record is substantially incomplete, or the reconstructed divergence estimates are inaccurate. Recently, a phylogenomic analysis [14] has reconstructed the youngest molecular estimates to date, and in general, reconstructed ages for the origin of Placentalia are decreasing with each published study [15]. These reconstructions are, however, still older than the earliest crown-group fossils, and contradict the conclusions of several fossil-based analyses, which have supported an origin of placentals close to the K–Pg boundary [7,16].

Molecular divergence estimates are necessarily informed by well-resolved fossil taxa. Owing to an historic lack of resolution in the higher level phylogeny of placental mammals, calibration points tend to be deeply nested within Placentalia. Even in the recent phylogenomic analysis by dos Reis *et al.* [14], calibration points were concentrated within Euarchontoglires, with 14 of 27 fossil calibration points within that superorder. Moreover, fossil occurrences provide only minimum age estimates for clades containing that taxon; the clade is unlikely to be exactly as old as the oldest fossil member of the clade. Indeed, the conclusion of O'Leary *et al.* [16] that Placentalia originated 64.85 Ma has been criticized as relying on ‘unjustified’ methods for dating internal nodes of the phylogeny [17]. A further consideration when assessing the timescale of placental evolution is the impact of evolutionary rate [18].

Despite the popular conception that the K–Pg mass extinction resulted in an explosive radiation, the evidence for changing evolutionary parameters at the K–Pg boundary is mixed. Several studies of mammals have found no difference between the latest Cretaceous and the earliest Palaeocene in either lineage accumulation rate [9,19] or body size evolution [20], a result consistent with molecular analyses of birds and acanthomorph teleosts [21,22]. Others have identified shifts in rate and mode of body size evolution [23] as well as mean body size [24] at the K–Pg boundary. A study of a single, well-sampled locality demonstrated that rate of per-lineage extinction increased in the latest Cretaceous, before an increase in per-lineage origination rates in the Palaeocene [25]. With the exception of this last example, few studies have included Palaeocene taxa as a large proportion of the data, despite these taxa being those that contributed overwhelmingly to the putative adaptive radiation. Moreover, analyses of evolutionary rates typically consider only single characters, risking exclusion of potentially important morphological change, whereas an extensive literature has demonstrated that multivariate approaches better capture the complexities of morphological evolution (e.g. [26–28]).

Here, we present a fossil-based analysis of the effect of the K–Pg mass extinction on placental evolution. We use the most recent stochastic techniques [29] to date phylogenetic trees [13] generated from a dataset of mostly Cretaceous and Palaeogene eutherians (electronic supplementary material, file S7), and reconstruct evolutionary rates in cladistic characters to assess change across a broad suite of morphological traits. Combined, these analyses answer two major questions in placental mammal evolution: when did Placentalia originate, and did the K–Pg mass extinction result in an Early Palaeogene adaptive radiation of placental mammals?

## 2. Material and methods

All analyses used R [30] code (electronic supplementary material, file S8), and stage-level time bins and taxon ranges for dating the phylogeny (electronic supplementary material, tables S1 and S2).

### (a) Tree selection

A recent phylogeny ([13], electronic supplementary material, file S2) is the largest to date to focus on Palaeogene and Cretaceous eutherians. There are many advantages of using trees from such a study for analysis of macroevolutionary patterns. Most importantly, the taxa sampled are proximal in time to the extinction event, meaning that rates of change are measured semi-directly, rather than inferred over tens of millions of years of subsequent evolution, as in the case of a tree containing only extant taxa. In taxa separated by tens of millions of years of evolution, convergent evolution driven by selection for similar characters adapted to a particular niche is more likely to be a problem than in taxa less separated in time from their ancestors. By sampling early members of clades, which are by definition closer to the last common ancestor they share with their sister taxon, informative characters that result from a shared evolutionary heritage are less likely to have been lost. Further, by including members of extinct clades, any effect of the extinction event can be measured across Eutheria, that is, avoiding the omission of those groups (such as Leptictida) that survived for several more epochs before going extinct. Using primarily extant taxa in reconstructing the past results in a somewhat tautological conclusion—there was no increase in extinction rates in lineages which did not go extinct—and risks biasing interpretation of ancient events by only considering taxa whose descendants happen to exist in the arbitrarily distant future that we call the present. Lastly, the trees each derive from a single analysis of 177 diverse genera, evenly sampling approximately two-thirds of Cretaceous and Palaeocene eutherian families, thereby avoiding loss of phylogenetic signal and lack of resolution.

Here, we analyse an initial sample of 564 most parsimonious trees (MPTs) derived from six phylogenetic analyses under varying data treatments and levels of constraint to well-supported relationships among extant orders ([13], see also the electronic supplementary material, file S2). The molecular-morphological scaffolds used in that analysis allowed the testing of various hypotheses of relationships of Palaeocene taxa to both extant orders and Cretaceous groups, such as competing hypotheses for the position of the enigmatic taxon *Purgatorius*. The topologies that resulted from those analyses differed in the patterns of relationships of some families, although some patterns were unambiguous, such as the absence of Cretaceous eutherians that were part of crown-group Placentalia [13]. We here use all generated trees from those analyses with different constraint levels to determine whether the results are robust to variation in topologies generated from these analyses. Further, as many tree lengths are to one or more decimal places, suboptimal topologies within a full step of the MPTs were also used to test over an even wider variety of plausible evolutionary relationships.

### (b) Dating the phylogenies

We dated the trees using a stochastic method, ‘cal3’ [29] which requires calculation of three rates: sampling (the per-time probability of sampling a taxon), diversification (the rate of origin of taxa) and extinction, (the rate at which taxa disappear from the fossil record). This approach is significantly better than alternative methods typically used in divergence date estimation and time-calibrating phylogenies for morphological and fossil-based datasets [31]. Cretaceous stages and Cenozoic North American Land Mammal Ages (NALMAs) were used as time bins. This difference reflects the geographical bias of Palaeogene mammals in favour of North America. By using NALMAs, uncertainty in first and last appearance dates of individual genera can be addressed while minimizing error introduced by taxa known from stratigraphic bounds that do not wholly overlap with the defined bins. We assigned first and last appearance bins to each taxon, and analysed the strict consensus of each set of trees.

When dating the trees, polytomies were resolved randomly. Testing several topologies derived from multiple analyses permitted a test of sensitivity of the results to any points of uncertainty in the placental phylogeny. In the suboptimal sets of trees, high-level polytomies resulted in topologies absent from the original sets of trees, while in optimal topologies, polytomies were generally smaller and closer to the tips; randomization would in that case be expected to have little impact on any reconstructed macroevolutionary pattern.

The taxonomic sample fairly represents the eutherian fossil record of the Cretaceous and Palaeocene, but the larger number of Palaeocene taxa may result in overestimating the sampling intensity of the Cretaceous fossil record. In order to test the effect of sampling rate, calculations were rerun with an assumed sampling rate of 0.5% per million years ago. This is highly conservative; estimates of the completeness of the earliest Cenozoic mammalian fossil record have been about 40% for an interval length of one lineage million years (LMY) [32].

Speciation and extinction rates were assumed to be equal, though this is not necessarily the case in reality [33]. Extinction rates cannot be estimated with any certainty from ultrametric phylogenies [34], but rather than the inclusion of additional arbitrary constants in an analysis derived from a non-ultrametric tree, the null model must be that net speciation is zero. Moreover, speciation and extinction rates track one another across palaeontological timescales [35], and as a result, this assumption is justifiable. Adaptive radiations follow periods of elevated extinction [36], and the K–Pg boundary is associated with local increases in both speciation and extinction rates in placental mammals [25].

### (c) Calculating evolutionary rates

Most analyses of how rates of evolution change across phylogeny focus on a single character—typically continuous in nature, such as body size [27,37]. However, a single parameter may not be a good proxy for overall morphological change through time. If the K–Pg extinction event did not affect one character trait, that does not preclude radical changes in other traits. Body size is correlated with several life-history and ecological variables [38–41], but might not correlate with other morphological transitions. When attempting to understand the overall evolution of a group, it is perhaps more useful to assess rate of change of a broad suite of characters, continuous or discrete. This is especially true in adaptive radiations, where selection pressures act in many new directions. We therefore assess rates of evolution across the discrete characters used to generate the trees.

In discrete characters, there are typically only two states. A change either occurs, or does not, with no possible intermediate, making rate calculations problematic. While it may be possible to quantify the shape of a feature or measure, for example, the position of a foramen through time, the required sample sizes to overcome both gaps in the fossil record and intraspecific variation make this impractical. Moreover, those gaps may result in an apparent jump from one state to another, missing the crucial period of transition and preventing identification of the variation. Where discrete characters are cladistically ideal—changing only at a single node, representing an unambiguous synapomorphy of a subset of the tree—measuring rate of change of that character is impossible. Most characters exhibit some homoplasy, but as there can be only one change per branch per character under maximum parsimony, assessing rates in individual discrete characters is impractical. However, using numerous discrete characters allows simultaneous optimization of multiple transitions across the tree. Each character has a distribution of state changes; if these changes are summed for each branch, rate of evolution on any given branch can be estimated, defined as the number of state changes per LMY. Dated phylogenies allow time-binning of branches and measuring of rates of change through geological time.

We calculated rates of discrete character evolution in three ways, implementing methods described in Lloyd *et al.* [28] and formalized in the R package Claddis [42]. Rate of evolution was defined as the number of discrete character transitions per LMY. Character ordering and weights were preserved from the original cladistic analysis. The character optimization method has little effect on the degree of homoplasy in the tree [43], but ACCTRAN and DELTRAN place characters at extremes, disproportionately increasing rates of evolution in the stem or crown, respectively. To avoid this, we only use unambiguous character transitions. For each set of dated phylogenies—six sets of MPTs and six suboptimal sets within a step, totalling 12 sets—a sample of 50 trees was used to calculate evolutionary rates, giving a total sample of 600 dated phylogenies.

Rates of individual branches were compared with a null model of equal rate of evolution across the tree, identifying branches with significantly high or low rates of evolution. The summed duration of all branches on the tree was calculated and considered to represent a continuum between zero and one; each branch was assigned some percentage of that continuum in proportion to temporal duration. Randomly determined values between zero and one were drawn, with the same number of repetitions as optimized character transitions. Each randomly drawn number represents an expected state change. In the null model, the number of character transitions on a branch is proportional to the duration of that branch.

This procedure was repeated 1000 times. If a branch had more observed character state transitions than predicted under the null model in at least 95% of repetitions, then that branch was considered to have significantly high rates. We identified branches with significantly high rates, and nodes subtending clades that differed significantly in rate, at which an intrinsic shift in background evolutionary rate can be inferred.

Finally, rates of evolution were compared among the previously mentioned time bins. Median rates of branches passing through each time bin were calculated. Owing to some short branch lengths calculated by the dating analysis, medians were preferred to means, which are sensitive to outliers. Median absolute deviation was calculated as a measure of error.

## 3. Results

### (a) Dating the origin of Placentalia

The distribution of divergence estimates for Placentalia consisted of mostly Late Cretaceous dates (electronic supplementary material, figure S1; table 1), with 95% of values falling between 65.52 and 69.53 Ma, and 69.37% of divergence dates older than 66 Ma. A Cretaceous origin of Placentalia is contrary to the conclusions of O'Leary *et al.* [16], and in agreement with most recent molecular estimates [14,17,19]. However, over 30% of reconstructed divergences are Palaeocene in age, while the Cretaceous estimates are still younger than those of previous molecular analyses [9,10,14] by 20–40 Ma. We conclude on the basis of these results that the best supported dates for the origin of Placentalia are Late Cretaceous.

**Table 1.** Dates and significances of clade origination. Divergence dates and 95% confidence intervals for major clades within and including Placentalia, each reconstructed from 6000 dated phylogenies representing six different constraint topologies. Suboptimal topologies are reconstructed as older due to the random resolution of polytomies which exist across Eutheria. Atlantogenata, Afrotheria and Xenarthra were reconstructed as originating in the Palaeogene at an alpha level of 0.05. The majority of divergences for Placentalia were reconstructed as Cretaceous. All values in millions of years before present.

	optimal median	optimal confidence intervals	suboptimal median	suboptimal confidence intervals	% optimal divergences in Cretaceous
Afrotheria	59.69	58.06–62.41	60.16	41.79–108.20	0
Xenarthra	60.46	58.82–65.35	61.68	58.97–108.84	1.1
Atlantogenata	60.46	58.82–65.35	61.71	58.89–108.84	1.1
Laurasiatheria	65.92	65.48–66.97	67.18	65.53–109.00	35.03
Euarchontoglires	65.45	64.01–66.67	65.74	65.01–104.82	13.53
Boreoeutheria	66.00	65.55–67.90	68.23	65.68–109.81	50.07
Placentalia	66.27	65.63–69.52	75.41	65.74–112.02	69.37

The divergence estimates for Boreoeutheria (Laurasiatheria plus Euarchontoglires) and Laurasiatheria were also extremely close to the End-Cretaceous mass extinction, with half of divergence estimates for Boreoeutheria falling either side of the Cretaceous–Palaeogene boundary (electronic supplementary material, figure S1; table 1). The distribution of Euarchontoglires divergences was primarily (86.47%) in the Palaeocene, and all other placental divergences were unambiguously reconstructed as Cenozoic. Results were independent of tree topology or sampling rate, although some suboptimal trees included large polytomies, resulting in a very wide distribution of divergence date estimates (table 1). Although the crown-group Placentalia most likely originated in the Cretaceous, most of the internal divergences at the ordinal level and below were Early Palaeocene in age.

#### (i) Rates of evolutionary change

**Figure 1.**
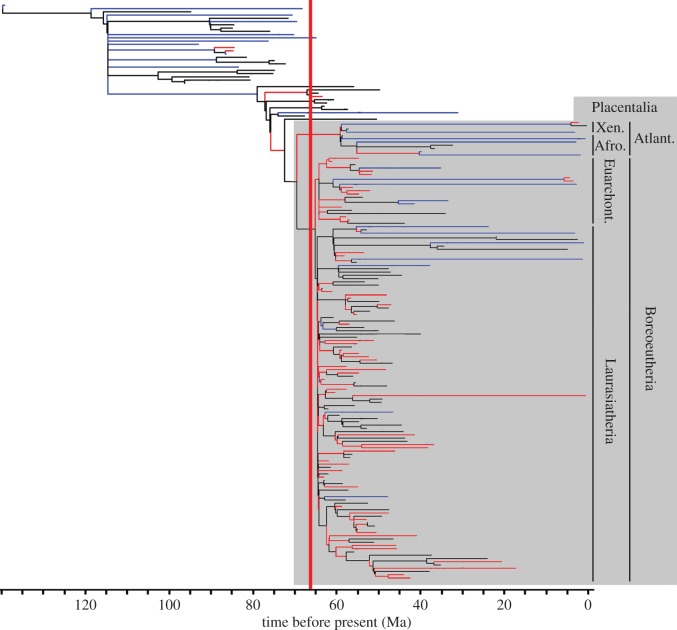
Randomly selected exemplar time-scaled phylogeny with branches coloured according to whether they have significantly lower (blue) or higher (red) evolutionary rates than would be expected given an equal rate model. The grey box indicates Placentalia, with the four placental superorders identified, while nodes and branches outwith the grey box are non-placental eutherians.

A uniform rate of evolution across the tree was consistently rejected; in all 600 sampled trees, more state changes occurred on the branch leading to Placentalia than would be expected by chance. With the exception of the origin of Zalambdalestidae in the Early Cretaceous, all branches with increased rates were associated either with Placentalia or on proximal branches leading to Placentalia (figure 1).

**Figure 2.**
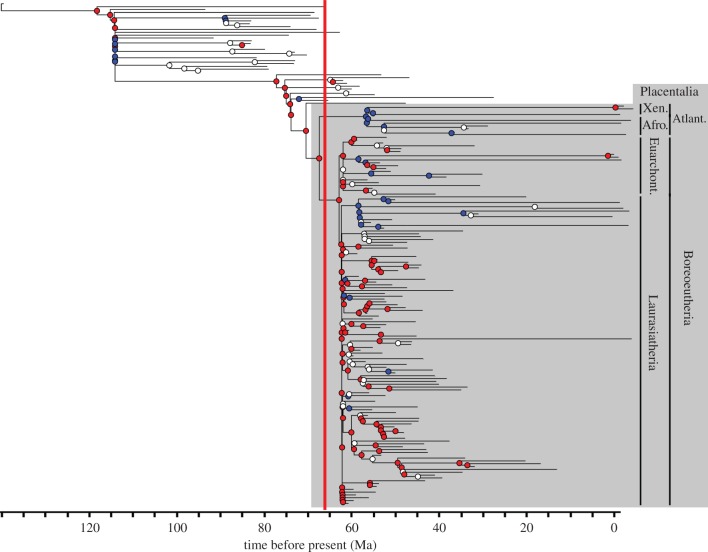
Randomly selected exemplar time-scaled phylogeny with nodes coloured according to whether the clade for which they are the last common ancestor has significantly lower (blue) or higher (red) evolutionary rates than the remainder of the tree. The grey box indicates Placentalia, with the four placental superorders identified, while nodes and branches outwith the grey box are non-placental eutherians.

**Figure 3.**
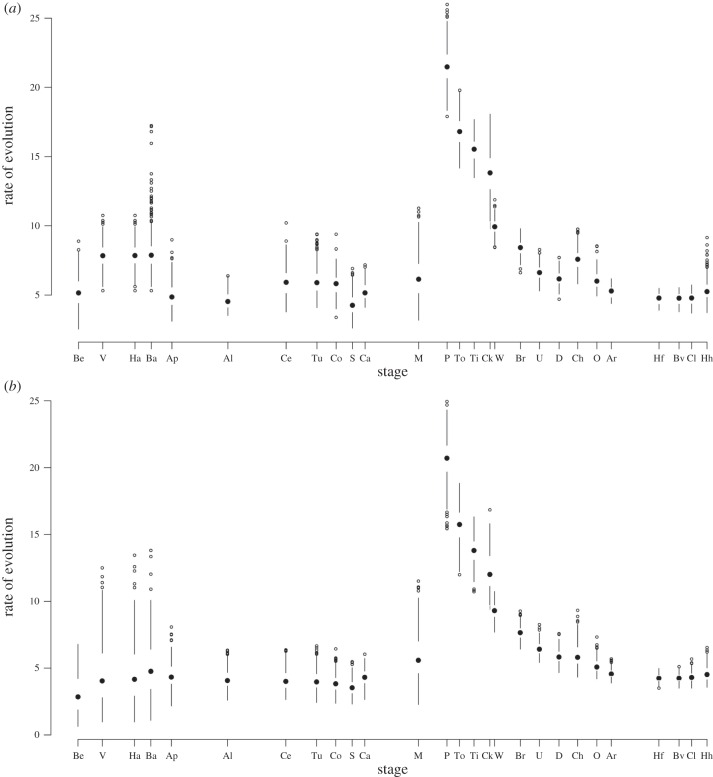
Two graphs depicting evolutionary rate through time calculated from the consensus of optimal topologies only from the six different original phylogenetic analyses. The Cretaceous–Palaeogene boundary falls between the Maastrichtian (M) stage and the Puercan (P) North American Land Mammal Age. (*a*) A sampling rate of 85%, which implies that the fossil record is relatively good throughout the tree. (*b*) A conservatively poor rate of 0.5%. Both show low Cretaceous rates with a significant two- to fourfold increase at the end-Cretaceous mass extinction, although (*b*) shows greater variance.

Placentalia was found to have a significantly higher intrinsic rate of evolution than the rest of the tree, as were several proximal clades encompassing Placentalia, including all nodes from Placentalia to the last common ancestor of the most basal cimolestids and Placentalia (figure 2). Within the crown, Atlantogenata and Euarchontoglires were notable exceptions with reduced evolutionary rates, implying that the increase in evolutionary rates in the Early Palaeogene is driven by the Laurasiatherian radiation. Early Palaeogene—particularly Palaeocene—time bins had significantly higher rates than Cretaceous bins (figure 3). Rates in the Cretaceous and after the Eocene were significantly lower than expected under an equal rate model.

## 4. Discussion

Although the diversification of Placentalia likely began in the latest Cretaceous, the majority of intraordinal diversification of placental mammals was in the Palaeogene. The origin of Placentalia is here reconstructed at least 15 Myr younger than the youngest estimate from molecular data [14], but older than recent fossil-based estimates [16].

The enigmatic genus *Protungulatum* has been considered important in dating the placental origin, although it is unclear whether it represents a placental [16] or a more basal eutherian [44]. Where it is supported as a placental, a Cretaceous origin for Placentalia is assured, as *Protungulatum* is known from the Maastrichtian. Here, however, all topologies place *Protungulatum* on the placental stem; no Mesozoic mammal is recovered as a crown placental in any supported tree consistent with the four clade model of placental interrelationships [13]. The date estimated here for the divergence of *Protungulatum* and Placentalia is approximately 75 Ma–9 Myr before the K–Pg boundary, and 6 Myr before the maximum estimated date of the origin of placental mammals. The lack of unambiguous Cretaceous placentals leaves a maximum gap of approximately 3 Myr between the origin of Placentalia and the first uncontentious placental fossils in the earliest Palaeogene [45], much smaller than those implied by molecular-derived dates. The age of Placentalia as reconstructed by Bayesian and clock-like methods is sensitive to evolutionary rate, with a predicted 10–20 fold increase in morphological evolutionary rate [18] required to reconcile the fossil record with typical molecular-derived dates. In the estimated 9 Myr between the divergence of Placentalia from *Protungulatum* and the K–Pg boundary, per-lineage rates of origination remained constant. In the earliest Palaeocene, species origination rates increased (electronic supplementary material, figure S2) following a high per-lineage extinction rate, consistent with local-scale patterns [25].

Alongside this increase in per-lineage speciation rate is an increase in the rate of morphological evolution. While Maastrichtian rates of evolution are higher than earlier Cretaceous bins, the maximum observed rates of discrete character evolution occurred in the earliest Palaeocene. This pattern is consistent with a recent phylogenomic network analysis on the early radiation of birds [46], which found that most internal diversification of crown Aves occurred rapidly at the beginning of the Palaeocene, and supports a Palaeocene radiation of placental mammals. That we find the origin of Placentalia to likely have been during the Late Cretaceous, this does not preclude the effect of the end-Cretaceous mass extinction on placental mammal evolution from being significant. Despite some divergences, having already taken place, the majority of diversification within Placentalia did not occur until after the mass extinction event. Moreover, what is now crown-group Placentalia was not the only clade to have undergone some diversification in the aftermath of the K–Pg boundary, as several closely related clades to the crown group—Leptictida and Cimolestidae in particular—also survived well into the Cenozoic. Our results strongly suggest that diversification had begun in the Late Cretaceous, but greatly accelerated in the Early Palaeocene, leading rapidly to increased taxonomic richness of eutherians in the beginning of the Cenozoic.

The eutherian mammals of the earliest Cenozoic also occupied a greater range of morphologies than Cretaceous eutherians [47]. Combined with the evidence from the current study that rates of speciation and character evolution increased after the end-Cretaceous mass extinction, this observation suggests that the placental mammal diversification was associated with rapid divergent evolution into a number of distinct morphologies. The novel morphologies observed in the earliest Cenozoic represent dental adaptations to new diets and increases in body size variation [48]. From a combination of high speciation rates, high rates of divergent morphological evolution, increases in disparity, and novel ecological modes of life, it is reasonable to characterize the placental diversification as an adaptive radiation.

Poor sampling of fossil data can affect reconstructions of divergence dates, and it is expected that a lower sampling intensity would result in greater uncertainty in dating the origin of Placentalia. When a conservatively low sampling rate of 0.5% per million year was applied, node divergence estimates were older, but not significantly different from estimations assuming higher sampling rates of 84%. Even with an estimated sampling rate twice that which has been calculated [32], the early diverging branches of Placentalia are pushed back into the Cretaceous. That the results are relatively similar across different sampling regimes suggests that sampling is adequate for accurately reconstructing early placental divergences. Indeed, sampling intensity had very little impact on reconstructed divergence dates for higher clades, implying that speciation and extinction rates are more important for estimating divergence times.

Thus, the inclusion here of Cretaceous and Palaeocene mammals, from which more accurate diversification rates could be determined, supports a later origination of Placentalia. Sampling of Cretaceous and Palaeocene eutherian mammals is relatively high in this analysis; 67% of Cretaceous and 62% of Palaeocene families for which fossils are known are represented, more than any analysis to date. Insofar as the fossil record is a reliable indicator of diversity, the rapid Palaeogene diversification represents a real event in placental evolution, supporting previous suggestions of high early rates in body size evolution [49].

Most of the Cretaceous displayed significantly lower rates than expected, probably because of an elevated estimation of background rate due to the extreme deviation observed in the Palaeocene. Evolutionary rates decline through the Eocene, and post-Eocene rates are significantly lower than those of the Palaeocene, but this may be due to reduction in taxonomic sampling: the post-Eocene sample is restricted to extant orders represented by only a few taxa, masking later radiations. By contrast, Cretaceous mammals are not undersampled; fewer taxa here are an accurate reflection of the lower taxic richness of Mesozoic eutherians [50]. These results are found across all topologies tested, indicating that both a Cretaceous origin of Placentalia and early high rates are well supported regardless of interordinal relationships.

To explain the missing Mesozoic placentals, the initial Late Cretaceous diversification might have occurred in some as yet unsampled ecosystem or region of the world [51]. This hypothesis is difficult to assess without analysing the completeness of the eutherian fossil record, or extensive sampling of other regions where basal placental (or derived non-placental eutherian) mammals might have diversified, such as the comparatively undersampled Gondwanan continents. India has to date yielded eutherians, but no placentals [6], while the earliest African placental is the basal afrothere *Ocepeia* from the Middle Palaeocene [52]. Cretaceous Madagascan mammals include gondwanatheres and multituberculates [53–55], but no eutherians. Only dryolestoids and gondwanatheres are known from the Late Cretaceous of South America [56,57], and the oldest placental mammals in Australia and Antarctica are Eocene [58,59]. It has been suggested that the abrupt faunal turnover at the K–Pg boundary is better explained by most new species being immigrants rather than representing new taxa [25], as there are no clear ancestors of taxa such as periptychid ‘condylarths’ in the preceding strata. Given the remarkable continuity of the San Juan Basin over this period [60], either morphological evolution was too rapid to be captured on geological timescales, or a significant bias exists in preservation or collection of Cretaceous mammals. However, sampling of the Cretaceous record would have to be orders of magnitude worse than the Palaeogene for there to be no recovered Mesozoic placental mammals [8].

Including Palaeocene taxa in analyses of the K–Pg mass extinction is needed to accurately reconstruct the evolutionary patterns of that interval and the processes that shaped the Early Cenozoic biota. The results of this analysis demonstrate the indispensable utility of fossils in reconstructing past events. The evidence from morphology and phylogeny suggests that the origin of Placentalia did not uniquely drive increased evolutionary rates for this clade—internal ordinal and superordinal radiations show much higher rates. In addition, these results demonstrate that the K–Pg mass extinction immediately preceded a dramatic increase in evolutionary rate in eutherian mammals, consistent with the hypothesis that this event was a significant driver of an Early Palaeogene adaptive radiation of placentals.

## Supplementary Material

Figure S1

## Supplementary Material

Figure S2

## Supplementary Material

Table S1

## Supplementary Material

Table S2

## Supplementary Material

File S1 - CF

## Supplementary Material

File S2 - CM

## Supplementary Material

File S3 - CP

## Supplementary Material

File S4 - DF

## Supplementary Material

File S5 - DM

## Supplementary Material

File S6 - DP

## Supplementary Material

File S7 - Discrete character-taxon matrix

## Supplementary Material

File S8 - R Code

## Data accessibility

Data and R code from this study have been deposited in the Dryad data repository at the following location: http://dx.doi.org/10.5061/dryad.0nr5s.
