# Supplementary figures and images for: Eutherians experienced elevated evolutionary rates in the immediate aftermath of the Cretaceous–Palaeogene mass extinction
Source: Proc Biol Sci. 2016 Jun 29;283(1833):20153026. doi: 10.1098/rspb.2015.3026 (PMC4936024; doi:10.1098/rspb.2015.3026)

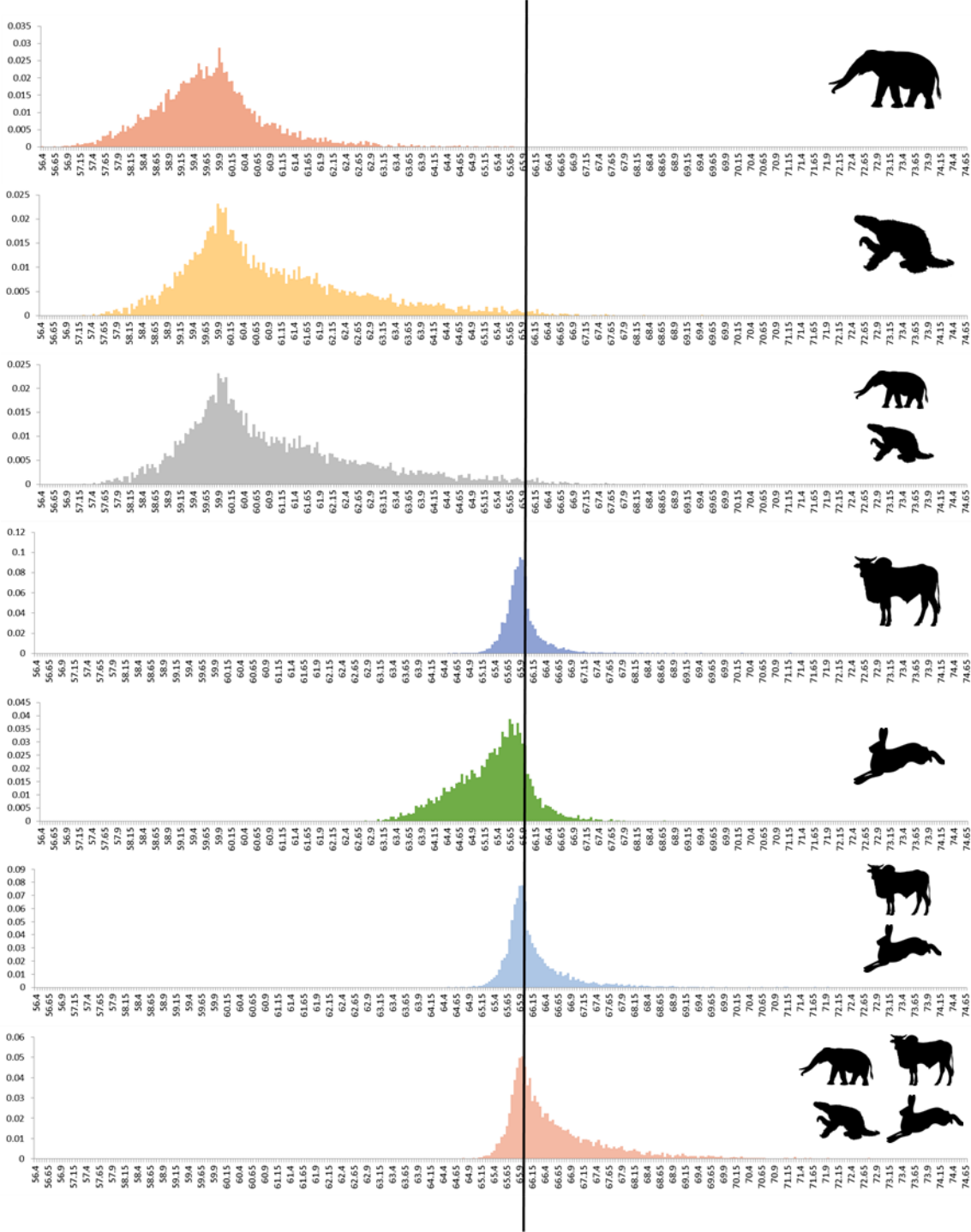

Supplement: Figure S1 [file rspb20153026supp1.pdf]

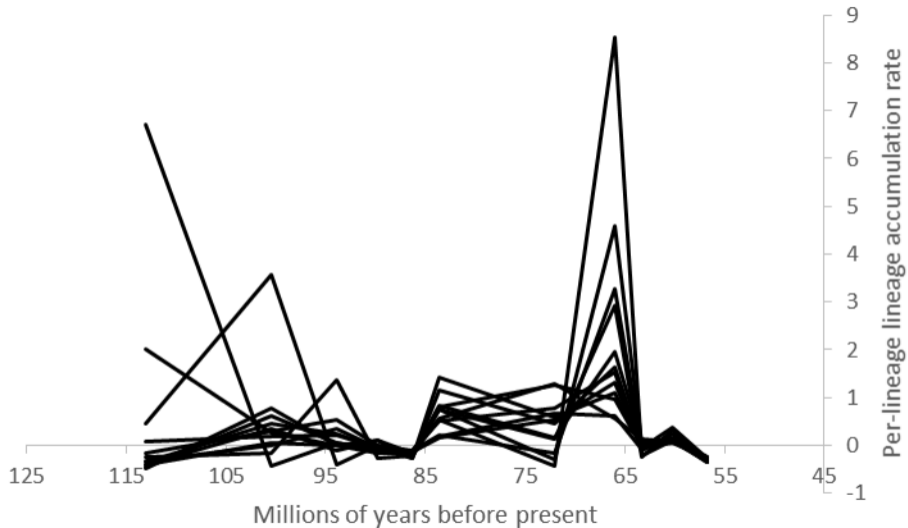

Supplement: Figure S2 [file rspb20153026supp2.pdf]
