# Supplementary material for: Eutherians experienced elevated evolutionary rates in the immediate aftermath of the Cretaceous–Palaeogene mass extinction
Source: Proc Biol Sci. 2016 Jun 29;283(1833):20153026. doi: 10.1098/rspb.2015.3026 (PMC4936024; doi:10.1098/rspb.2015.3026)
Supplement: Table S1 [file rspb20153026supp3.pdf]

Table S1 – Dates of the Cretaceous geological stages and Cenozoic North American Land Mammal Ages as used for dating the topologies and determining taxon occurrences.

STAGE	START TIME	END TIME	STAGE	START TIME	END TIME
**BERRIASIAN**	145	139.8	**TIFFANIAN**	60.2	56.8
**VALANGINIAN**	139.8	132.9	**CLARKFORKIAN**	56.8	55.8
**HAUTERIVIAN**	132.9	129.4	**WASATCHIAN**	55.8	50.3
**BARREMIAN**	129.4	125	**BRIDGERIAN**	50.3	46.2
**APTIAN**	125	113	**UINTAN**	46.2	42
**ALBIAN**	113	100.5	**DUCHESNEAN**	42	38
**CENOMANIAN**	100.5	93.9	**CHADRONIAN**	38	33.9
**TURONIAN**	93.9	89.8	**ORELLAN**	33.9	30.8
**CONIACIAN**	89.8	86.3	**ARIKAREEAN**	30.8	20.6
**SANTONIAN**	86.3	83.6	**HEMINGFORDIAN**	20.6	16.3
**CAMPANIAN**	83.6	72.1	**BARSTOVIAN**	16.3	13.6
**MAASTRICHTIAN**	72.1	66	**CLARENDONIAN**	13.6	10.3
**PUERCAN**	66	63.3	**HEMPHILLIAN**	10.3	4.9
**TORREJONIAN**	63.3	60.2	**BLANCAN TO RECENT**	4.9	0
