# Supplementary material for: Eutherians experienced elevated evolutionary rates in the immediate aftermath of the Cretaceous–Palaeogene mass extinction
Source: Proc Biol Sci. 2016 Jun 29;283(1833):20153026. doi: 10.1098/rspb.2015.3026 (PMC4936024; doi:10.1098/rspb.2015.3026)
Supplement: Table S2 [file rspb20153026supp4.pdf]

Table S2 – Occurrences of each genus in this analysis in the time bins from Table 1. Stage 1 is the Berriasian, Stage 12 the Maastrichtian, Stage 13 the Puercan, and so on.

TAXON	FIRST STAGE	LAST STAGE	TAXON	FIRST STAGE	LAST STAGE
Peramus	1	1	*Mimatuta*	13	13
Deltatheridium	11	12	*Desmatoclaenus*	13	15
Sheikhdzheilia	6	7	*Protoselene*	13	15
Avitotherium	11	11	*Bunophorus*	17	18
Gallolestes	11	11	*Diacodexis*	17	18
Alostera	11	12	*Homacodon*	18	20
Parazhelestes	9	9	*Hyopsodus*	16	20
Aspanlestes	9	11	*Meniscotherium*	17	17
Zhelestes	8	9	*Phenacodus*	14	18
Paranyctoides	8	12	*Macrocranion*	15	20
Batodon	11	12	*Alsaticopithecus*	18	18
Maelestes	11	11	*Teilhardimys*	15	18
Bobolestes	6	7	*Apheliscus*	15	17
Bulaklestes	9	9	*Haplomylus*	15	19
Daulestes	8	9	*Hilalia*	18	18
Uchkudukodon	9	9	*Orthaspidotherium*	15	15
Kennalestes	9	11	*Pleuraspidotherium*	15	15
Asioryctes	11	11	*Poebrotherium*	21	23
Ukhaatherium	11	11	*Gobiohyus*	18	21
Kulbeckia	9	10	*Leptomeryx*	21	23
Lainodon	11	11	*Elomeryx*	21	24
Zhangolestes	5	7	*Indohyus*	18	20
Barunlestes	11	11	*Pakicetus*	17	19
Alymlestes	11	11	*Rodhocetus*	19	19
Zalambdalestes	11	11	*Aphronorus*	13	16
Cimolestes	11	15	*Pentacodon*	14	15
Puercolestes	13	13	*Conacodon*	13	13
Betonnia	13	13	*Anisonchus*	13	15
Eoryctes	17	17	*Periptychus*	13	15
Chacopterygus	13	13	*Ectoconus*	13	13
Procerberus	13	14	*Hemithlaeus*	13	13
Purgatorius	13	15	*Esthonyx*	16	18
Protungulatum	12	13	*Alcidedorbignya*	13	13
Gypsonictops	11	12	*Coryphodon*	16	18
Leptictis	20	22	*Pantolambda*	14	15
Prodiacodon	13	17	*Titanoides*	15	16
Asiostylops	14	14	*Cyriacotherium*	15	16
Arctostylops	15	16	*Lambdotherium*	17	18
Chaetophractus	26	28	*Litolophus*	16	16
Utaetus	15	15	*Molinodus*	13	13

Bradypus	28	28	*Haploconus*	13	14
Tamandua	28	28	*Tetraclaenodon*	14	15
Procavia	27	28	*Copecion*	15	18
Eritherium	15	15	*Ectocion*	14	18
Potamogale	28	28	*Eohippus*	17	17
Adapisorex	14	16	*Hyracotherium*	17	17
Rhynchocyon	23	28	*Homogalax*	17	17
Chambius	16	20	*Heptodon*	17	17
Todralestes	16	18	*Oxyclaenus*	13	14
Dilambdogale	21	21	*Loxolophus*	13	14
Widanelfarasia	22	22	*Eoconodon*	13	13
Lessnessina	17	17	*Goniacodon*	13	15
Tribosphenomys	15	16	*Hapalodectes*	15	18
Paramys	16	21	*Ankalagon*	14	14
Rhombomylus	17	17	*Pachyaena*	15	17
Gomphos	17	17	*Dissacus*	13	17
Ptilocercus	28	28	*Mesonyx*	18	19
Tupaia	25	28	*Sinonyx*	16	16
Cynocephalus	28	28	*Arctocyon*	14	15
Elpidophorus	14	15	*Claenodon*	14	15
Worlandia	16	16	*Anacodon*	15	18
Plagiomene	16	17	*Thryptacodon*	14	17
Bisonalveus	15	15	*Chriacus*	13	17
Notharctus	17	19	*Onychonycteris*	17	17
Adapis	20	21	*Icaronycteris*	16	18
Apatemys	17	21	*Didymictis*	15	19
Mixodectes	13	15	*Viverravus*	15	19
Elphidotarsius	14	15	*Protictis*	14	19
Cantius	17	19	*Uintacyon*	15	19
Saxonella	15	15	*Vulpavus*	15	20
Lambertocyon	15	16	*Miacis*	17	21
Escavadodon	14	14	*Wyolestes*	17	17
Onychodectes	13	13	*Prolimnocyon*	16	18
Didelphodus	17	20	*Pyrocyon*	17	17
Acmeodon	14	15	*Dipsalidictis*	16	17
Gelastops	13	15	*Tytthaena*	15	16
Solenodon	28	28	*Bessoecetor*	14	15
Parapternodus	17	17	*Paleosinopa*	15	18
Leptacodon	13	17	*Pararyctes*	13	17
Wyonycteris	16	17	*Paleoryctes*	13	16
Litocherus	14	15	*Aaptoryctes*	15	16
Centetodon	17	23	*Eurotamandua*	18	18
Blarina	28	28	*Palaeanodon*	16	17
Tubulodon	17	17	*Eomanis*	18	18
Domnina	19	23	*Pteropus*	28	28
Echinosorex	28	28	*Plesiadapis*	14	17

Uropsilus	28	28	*Simpsonotus*	15	15
Desmana	27	28	*Protolipterna*	15	15
Oreotalpa	21	21			
